# Supplementary material for: Identification of photoperiod-induced specific miRNAs in the adrenal glands of Sunite sheep (Ovis aries)
Source: Front Vet Sci. 2022 Jul 22;9:888207. doi: 10.3389/fvets.2022.888207 (PMC9354845; doi:10.3389/fvets.2022.888207)
Supplement: Supplementary file 1 [file Data_Sheet_1.ZIP › Supplementary file/miRDeep2 file.zip!/miRDeep2 file/Know_Structure/LP42A3/oar-mir-191.pdf]

miRBase precursor : oar-mir-191
Total read count : 103112
oar-miR-191 read count : 102859
remaining reads : 253

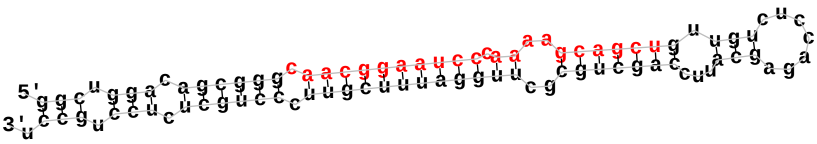

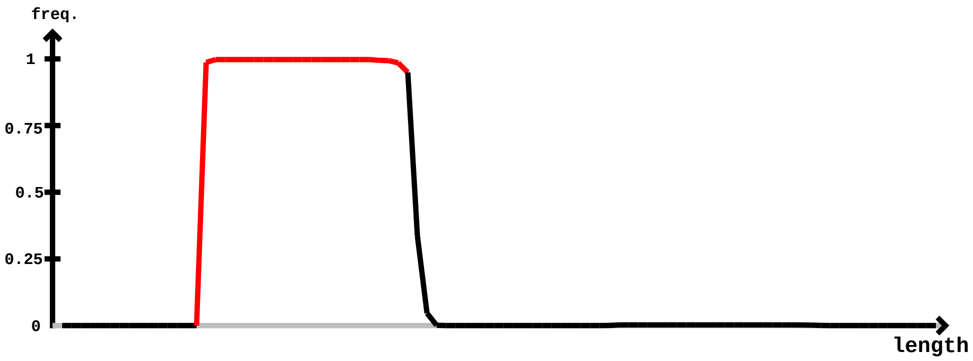


```
                oar-miR-191
5'- ggcuggacagcgggcaacggaaucccaaaagcagcuguugucuccagagcauuccagcugcgcuuggauuucguucccugcucuccugccu   -3'    exp
    (((.(((.((((((.((((((((((.((..(((((((.(((.......)))...))))))).)))))))))))).)))))).))).))).    reads       mm          sample
    ..............caacggaaucccaaaag............................................................    2           0           seq
    ..............caacggaaucccaaaagc...........................................................    310         0           seq
    ..............caacggaaucccaaGagc...........................................................    1           1           seq
    ..............caacggaaucccaaaagca..........................................................    125         0           seq
    ..............caacggaaucccaaaagcag.........................................................    850         0           seq
    ..............caacggaaucccaaaagcagc........................................................    3676        0           seq
    ..............caacggaaucccaaaagcagcu.......................................................    62879       0           seq
    ..............caacggaaucccaaaagcagcug......................................................    29395       0           seq
    ..............caacggaaucccaaaagcagcugu.....................................................    4394        0           seq
    ..............caacggaaucccaaaagcagcuguu....................................................    110         0           seq
    ..............caacggaaucccaaaagcagcuguugu..................................................    1           0           seq
    ...............aacggaaucccaaaagc...........................................................    1           0           seq
    ...............aacggaaucccaaaagcag.........................................................    1           0           seq
    ...............aacggaaucccaaaagcagc........................................................    17          0           seq
    ...............aacggaaucccaaaagcagcu.......................................................    353         0           seq
    ...............aacggaaucccaaaagcagcug......................................................    464         0           seq
    ...............aacggaaucccaaaagcagcugu.....................................................    267         0           seq
    ...............aacggaaucccaaaagcagcuguu....................................................    6           0           seq
    ................acggaaucccaaaagcagcu.......................................................    4           0           seq
    ................acggaaucccaaaagcagcug......................................................    1           0           seq
    .................cggaaucccaaaagcagcug......................................................    1           0           seq
    .......................cccaaaagcagcuguuC...................................................    1           1           seq
    .......................................................agcugcgcuuggauuucgu.................    1           0           seq
    .......................................................agcugcgcuuggauuucguuc...............    1           0           seq
    .......................................................agcugcgcuuggauuucguucc..............    1           0           seq
    ........................................................gcugcgcuuggauuucgu.................    1           0           seq
    ........................................................gcugcgcuuggauuucguu................    2           0           seq
    ........................................................gcugcgcuuggauuucguuc...............    10          0           seq
    ........................................................gcugcgcuuggauuucguucc..............    12          0           seq
    ........................................................gcugcgcuuggauuucguuccc.............    13          0           seq
    ........................................................gcugcgcuuggauuucguucccu............    1           0           seq
    .........................................................cugcgcuuggauuucguu................    5           0           seq
    .........................................................cugcgcuuggauuucguuc...............    10          0           seq
    .........................................................cugcgcuuggauuucguucc..............    32          0           seq
    .........................................................cugcgcuuggauuucguuccc.............    109         0           seq
```

oar-miR-191

ggcuggacagcggg**caacggaaucccaaaagcagcu**guugucuccagagcauuccagcugcgcuuggauuucguucccugcucuccugccu
....cugcgcuuggauuucguucccu............ 53 0 seq
....ugcgcuuggauuucguucccu............ 2 0 seq
